# Associations of sarcopenia with peak expiratory flow among community-dwelling elderly population: based on the China Health and Retirement Longitudinal Study (CHARLS)

**DOI:** 10.1007/s41999-023-00838-2

**Published:** 2023-07-19

**Authors:** Yun-Yun He, Mei-Ling Jin, Jing Chang, Xiao-Juan Wang

**Affiliations:** 1 Department of General Medicine, Beijing Chao-yang Hospital, Capital Medical University, Beijing, 100020 China; 2 Department of Nephrology, Beijing Chao-yang Hospital, Capital Medical University, Beijing, 100020 China

**Keywords:** Sarcopenia, Skeletal muscle mass, Hand grip strength, Physical performance, Peak expiratory flow, Community-dwelling elderly

## Abstract

**Purpose:**

To cross-sectionally and longitudinally investigate the correlations of sarcopenia and its components with peak expiratory flow (PEF) among Chinese community-dwelling elderly people.

**Methods:**

The data were extracted from the China Health and Retirement Longitudinal Study (CHARLS). A total of 4053 participants aged ≥ 60 years were enrolled from CHARLS 2011, and 2810 were followed up until 2015. Participants were classified into no-sarcopenia, non-severe sarcopenia, and severe sarcopenia groups based on skeletal muscle mass index (SMI), hand grip strength (HGS), and physical performance [gait speed, five-repetition chair stand test (5CST) and short physical performance battery (SPPB)]. Multivariate linear and logistic regression analyses were used to evaluate the associations of sarcopenia and its components with PEF cross-sectionally and longitudinally.

**Results:**

In the cross-sectional analysis, the prevalence of non-severe sarcopenia was 14.6% and severe sarcopenia was 4.9%. The results of linear regression analysis revealed that sarcopenia and its components were all correlated with PEF and PEF%pred. In the longitudinal analysis, compared with non-sarcopenia, subjects with severe sarcopenia were associated with a higher risk of PEF (OR = 2.05, 95%CI = 1.30–3.26) and PEF%pred (OR = 1.83, 95%CI = 1.17–2.86) decline. The changes in physical performance were correlated with changes in PEF and PEF%pred. No associations were observed between changes in SMI and PEF as well as PEF%pred.

**Conclusions:**

We demonstrated the associations of baseline sarcopenia status with PEF and longitudinal PEF decline. Also, the changes in physical performance were associated with changes in PEF during a 4-year follow-up. It indicates that improving sarcopenia, especially physical performance may increase PEF.

**Supplementary Information:**

The online version contains supplementary material available at 10.1007/s41999-023-00838-2.

## Key summary points

**Aim:**

To investigate the correlations of sarcopenia and its components with peak expiratory flow (PEF) among Chinese community-dwelling elderly people.

**Findings:**

The baseline sarcopenia status was related to PEF and PEF decline in Chinese community-dwelling elderly population by cross-sectional and longitudinal analysis. Also, the changes in physical performance were associated with changes in PEF during a 4-year follow-up.

**Message:**

Improving sarcopenia, especially physical performance may contribute to the increase of PEF.

**Supplementary Information:**

The online version contains supplementary material available at 10.1007/s41999-023-00838-2.

## Introduction

Sarcopenia is a geriatric syndrome characterized by loss of skeletal muscle mass plus loss of muscle strength and/or reduced physical performance [1]. According to the 2019 consensus of the Asian Working Group for Sarcopenia (AWGS), the prevalence of sarcopenia in Asia ranged from 5.5 to 25.7% [2]. In China, a recent meta revealed that the prevalence of sarcopenia in community-dwelling older adults was 12.9% in men and 11.2% in women [3]. What is more, the prevalence of sarcopenia increases with age. Sarcopenia in the elderly was usually occult in onset but could result in physical dysfunction. Previous studies have demonstrated that sarcopenia is related to various adverse events, including cardiovascular diseases, falls, disability, mortality, and so on [4–6].

Similarly to systemic skeletal muscle, aging could cause loss of respiratory muscle mass and respiratory muscle strength, and/or decline of pulmonary function, which is known as respiratory sarcopenia [7]. It is reported that low skeletal muscle mass and sarcopenia were related to poor pulmonary function in chronic obstructive pulmonary disease (COPD) [8]. A systematic review including twenty-three studies involving 9637 participants with COPD revealed that people with sarcopenia had lower predicted forced expiratory volume in the first second (FEV1) and poorer exercise tolerance and quality of life compared with those who did not [9]. A few cross-sectional studies also investigated the relationship between sarcopenia and pulmonary function in general adults without clinically apparent lung diseases, and had come to a similar conclusion [10–12]. Moreover, peripheral muscle strength such as hand grip strength may affect respiratory muscle strength [13].

The peak expiratory flow (PEF), a simple screening tool of pulmonary function, refers to the instantaneous velocity of expiratory flow when it is the fastest in the process of forced spirometry, and reflects the strength of the respiratory muscle [14]. Recently, some scholars proposed that among community-dwelling older people, the definition of respiratory sarcopenia based on peak expiratory flow was useful and correlated with conventional sarcopenia and long-term care insurance certification [15]. However, to our knowledge, few studies have investigated the correlation of sarcopenia and its components with PEF based on longitudinal studies.

Therefore, in this study, using data from the China Health and Retirement Longitudinal Study (CHARLS), we conducted a cross-sectional analysis to evaluate the association of sarcopenia with pulmonary function, as assessed via PEF. We also longitudinally examined the associations of the change trajectories between sarcopenic components with PEF over a 4-year follow-up among Chinese community-dwelling elderly people.

## Methods

### Study design and population

The data of this study were extracted from the CHARLS, an ongoing nationally representative longitudinal cohort survey targeting the Chinese population aged 45 years and older. Detailed information about the CHARLS design and methods has been previously described in detail [16, 17]. In brief, the CHARLS project conducted a national baseline survey in 2011–2012 by multistage probability-proportionate-to-size sampling technology and was followed up consecutively through face-to-face computer-assisted personal interviews. The baseline survey recruited 17,708 participants from 450 villages/residents and 150 counties/districts within 28 provinces in China. The follow-up surveys were conducted in 2013, 2015, and 2018. The CHARLS was approved by the Institutional Review Board of Peking University (IRB00001052-11015) and informed consent was obtained from all subjects.

**Fig. 1:**
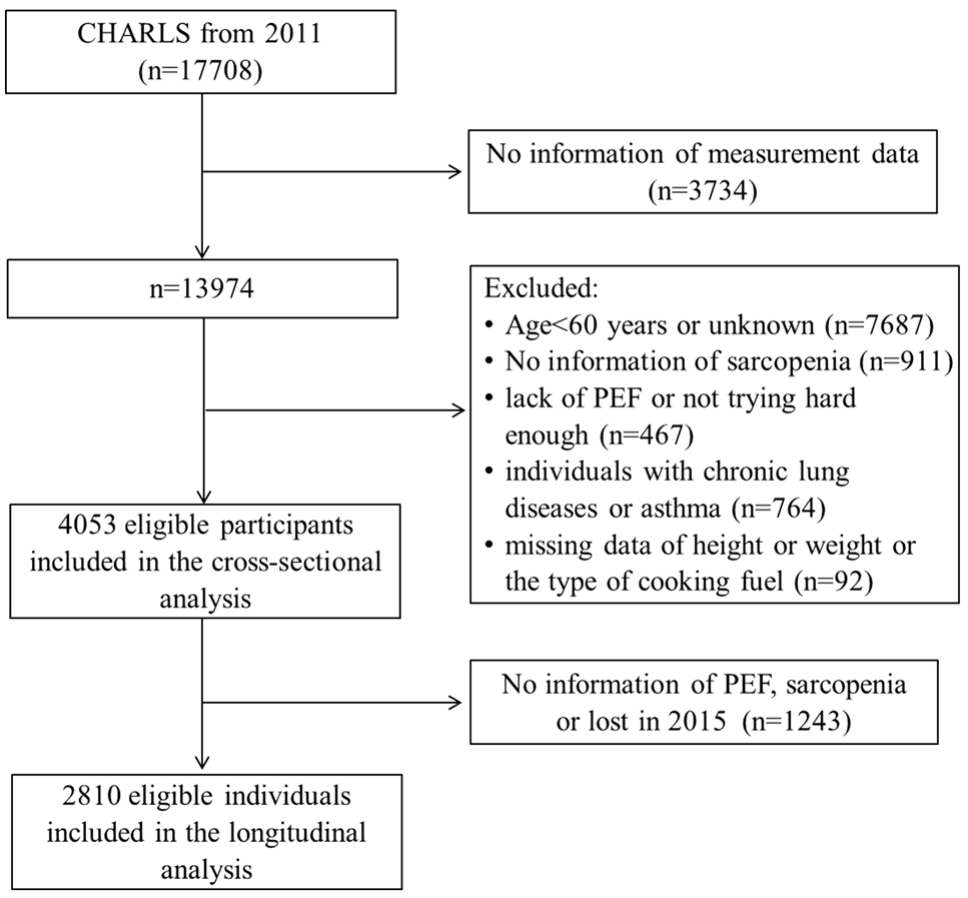
The detailed flowchart of this study

In this study, we obtained the baseline data from CHARLS 2011 and the followed-up information from 2015. The exclusion criteria at baseline were as follows: (1) no information of sarcopenia; (2) aged less than 60 years old; (3) individuals with a self-reported history of chronic lung diseases or asthma; (4) lack of PEF or not trying hard enough; and (5) missing data of height or weight or the type of cooking fuel. Finally, 4053 subjects were included in the baseline survey. During the follow-up wave, 1243 individuals lacked information of sarcopenia or PEF, or lost in 2015, leaving 2810 eligible participants for the final longitudinal analysis. The detailed flowchart of this current study is shown in Fig. 1.

### Covariates

The socio-demographic and health-related information on age, sex, educational level, smoking and drinking status, height, body weight (BW), marital status (married or widowed/divorced/single), residential area (urban or rural), type of cooking fuels, and self-reported comorbid diseases including hypertension, diabetes, heart problems, stroke, and kidney disease of all participants was collected by well-trained investigators through face-to-face interviews. The body mass index (BMI) was calculated by dividing the BW (in kilograms) by the height (in meters) squared. The education level was divided into elementary school or below, secondary school, and college or above. The smoking status was divided into never smokers, former smokers, and current smokers. The drinking was defined as drinking in the last year. Clean fuel for household cooking was defined as natural gas, marsh gas, liquefied petroleum gas, and electric, while solid cooking fuel referred to coal and crop residue/wood burning.

### Assessment of sarcopenia

The details about the definitions for sarcopenia components in the CHARLS have been described previously [18, 19]. The skeletal muscle mass was estimated by the appendicular skeletal muscle mass (ASM), which was calculated with a previously validated anthropometric equation in Chinese adults [20].

$$ {\text{ASM }} = 0.{193 } \times {\text{ BW }} + \, 0.{1}0{7 } \times {\text{ height }} - { 4}.{157 } \times {\text{ sex }} - 0.0{37 } \times {\text{ age }}{-}{ 2}.{631}{\text{.}} $$

Of this equation, the BW, height, and age were measured in kilograms, centimeters, and years, respectively. For sex, males were assigned the value of 1, and females were assigned 2. The skeletal muscle mass index (SMI) was calculated by ASM/height^2^. The cut-off value for defining low muscle mass was based on the sex-specific lowest 20% of the SMI among the study population [18, 21], with < 6.85 kg/m^2^ in men and < 4.98 kg/m^2^ in women.

Hand grip strength (HGS) was measured using the right or left hand with a dynamometer (Yuejian™ WL-1000, Nantong, China) in kilograms at a right angle (90˚) for a few seconds as hard as possible. Each hand was tested twice and the maximum value of the four attempts was extracted for the next analysis [22]. The cut-off points for low grip strength were defined as < 28.0 kg for men and < 18.0 kg for women.

Gait speed was calculated by the time to complete a 2.5-m course at a normal pace. The average of available values twice was used for statistical analysis. The five-repetition chair stand test (5CST) was assessed by the time taken to stand from a chair five times in seconds as quickly as possible [19]. Besides gait speed and 5CST, the assessment of short physical performance battery (SPPB) also included the balance tests including semi-tandem stands, full-tandem stands, and side-by-side stands. The summary score of SPPB was 12 points with 4 points for each physical performance measurement [23]. AWGS 2019 recommends defining low physical performance based on either SPPB ≤ 9, gait speed < 1 m/s, or 5CST ≥ 12 s.

Sarcopenia is defined as low muscle mass plus low muscle strength or low physical performance. Subjects with low muscle mass, low muscle strength, and low physical performance are considered severe sarcopenia.

### Ascertainment of PEF

PEF was measured using a peak flow meter (Everpure ™, Shanghai, China). The participants were asked to take a deep breath and place their lips around the mouthpiece, and then blow as hard and fast as possible. The process was repeated three times at 30-s intervals and the highest value was used in the next analysis.

The predicted PEF was calculated with previously validated equations by Zhong Nanshan in Chinese adults: 75.6 + 20.4 × age−0.41 × age^2^ + 0.002 × age^3^ + 1.19 × height for males and 282.0 + 1.79 × age−0.046 × age^2^ + 0.68 × height for females. Of the equations, age and height were measured in years and centimeters, respectively. The percentage of actual value to predicted PEF was expressed as PEF%pred. PEF%pred < 80% was defined as airflow limitation [24].

### Statistical analysis

All continuous variables of the study were shown as mean ± standard deviation. Statistical analysis of continuous variables was performed using an unpaired Student *t*-test between two groups and one-way analysis of variance followed by a least-squares difference test for multiple comparisons. Categorical variables were expressed as frequency and percentages, and performed by chi-square test. Multivariate linear regression analysis with enter method was performed to evaluate the cross-sectional associations of sarcopenia and its components with PEF, as well as the associations of the change trajectories between sarcopenic components with PEF from 2011 to 2015, respectively. Multivariate logistic regression analysis with enter method was used to investigate the associations between airflow limitation and sarcopenia, including its defining components. The same method was used to explore the incidence of PEF or PEF%pred decline according to baseline sarcopenia status in the longitudinal analysis. Three adjusted models were used in the multivariate linear and logistic regression analysis: model 1 unadjusted; model 2 adjusted for age and sex; model 3 adjusted for model 2 plus education level, smoking status, drinking status, BMI, marital status, residential area, type of cooking fuels, and comorbid diseases. In the longitudinal analysis, baseline PEF or PEF%pred was also added to model 3. The regression coefficients (β) and 95% confidence intervals (CI) were expressed by linear regression analysis, while odds ratios (OR) and 95% CI were computed by logistic regression analysis.

All tests were carried out using SPSS version 22.0 statistical software. Differences were considered statistically significant at a two-tailed *P* value < 0.05.

## Results

### Baseline characteristics of subjects

The baseline characteristics of enrolled subjects are listed in Table 1. A total of 4053 participants were included in the cross-sectional analysis. The mean age was 67.39 years old, and 50.4% of participants were men. Among these 4053 elderly, the prevalence of sarcopenia was 19.5%, of which, non-severe sarcopenia was 14.6% and severe sarcopenia was 4.9%. Compared to non-sarcopenia, the participants with sarcopenia were more likely to be older and single, live in rural, had a lower level of education, smoke, and use solid fuels for household cooking (all *P* < 0.01). The values of PEF and PEF%pred were significantly lower, while the proportion of airflow limitation was higher in sarcopenic subjects than in non-sarcopenia, especially in the severe sarcopenia group (all *P* < 0.01).

**Table 1:** Baseline characteristics of enrolled subjects

	Total (*n* = 4053)	Non-sarcopenia (*n* = 3263)	Non-severe sarcopenia (*n* = 592)	Severe sarcopenia (*n* = 198)	*P* value
Age (years)	67.39 ± 6.25	66.44 ± 5.63	70.53 ± 6.98	73.72 ± 6.89	< 0.001
Gender, *n* (%)					0.077
Male	2042 (50.4)	1646 (50.4)	283 (47.8)	113 (57.1)	
Female	2011 (49.6)	1617 (49.6)	309 (52.2)	85 (42.9)	
Marital status, *n* (%)					< 0.001
Married	3184 (78.6)	2639 (80.9)	426 (72.0)	119 (60.1)	
Widowed/divorced/single	869 (21.4)	624 (19.1)	166 (28.0)	79 (39.9)	
Residential area, *n* (%)					< 0.001
Urban	790 (19.5)	725 (22.2)	51 (8.6)	14 (7.1)	
Rural	3263 (80.5)	2538 (77.8)	541 (91.4)	184 (92.9)	
Education level, *n* (%)					< 0.001
Elementary school or below	3308 (81.6)	2597 (79.6)	524 (88.5)	187 (94.4)	
Secondary school	674 (16.6)	600 (18.4)	65 (11.0)	9 (4.6)	
College and above	71 (1.8)	66 (2.0)	3 (0.5)	2 (1.0)	
Smoking status					< 0.001
Never	2375 (58.6)	1955 (59.9)	319 (53.9)	101 (51.0)	
Former	404 (10.0)	339 (10.4)	44 (7.4)	21 (10.6)	
Current	1274 (31.4)	969 (29.7)	229 (38.7)	76 (38.4)	
Current drinking					0.879
No	2750 (67.9)	2209 (67.7)	407 (68.7)	134 (67.7)	
Yes	1303 (32.1)	1054 (32.3)	185 (31.3)	64 (32.9)	
Type of cooking fuels					< 0.001
Clean	1589 (39.2)	1369 (42.0)	167 (28.2)	53 (26.8)	
Solid	2464 (60.8)	1894 (58.0)	425 (71.8)	145 (73.2)	
Height (cm)	156.74 ± 8.73	157.48 ± 8.33	154.16 ± 9.69	152.19 ± 9.34	< 0.001
Weight (kg)	56.60 ± 10.96	59.67 ± 9.62	44.20 ± 5.91	43.03 ± 5.58	< 0.001
BMI (kg/m^2^)	22.96 ± 3.61	24.02 ± 3.13	18.56 ± 1.50	18.57 ± 1.70	< 0.001
Hypertension, *n* (%)	1238 (30.5)	1098 (33.7)	101 (17.1)	39 (19.7)	< 0.001
Diabetes or high blood sugar, *n* (%)	265 (6.5)	245 (7.5)	13 (2.2)	7 (3.5)	< 0.001
Heart problems, *n* (%)	546 (13.5)	471 (14.4)	53 (9.0)	22 (11.1)	0.001
Stroke, *n* (%)	109 (2.7)	95 (2.9)	8 (1.4)	6 (3.0)	0.095
Kidney disease, *n* (%)	228 (5.6)	191 (5.9)	26 (4.4)	11 (5.6)	0.389
HGS (kg)	30.18 ± 9.43	31.02 ± 9.50	29.35 ± 7.26	18.85 ± 5.80	< 0.001
Gait speed (m/s)	0.64 ± 0.23	0.66 ± 0.23	0.59 ± 0.21	0.52 ± 0.19	< 0.001
5CST (s)	11.66 ± 4.76	11.52 ± 4.84	11.64 ± 3.60	14.04 ± 5.87	< 0.001
SPPB	9.60 ± 1.97	9.73 ± 1.94	9.37 ± 1.85	8.17 ± 2.23	< 0.001
ASM (kg)	16.35 ± 4.16	17.06 ± 3.92	13.46 ± 3.91	13.29 ± 3.66	< 0.001
SMI (kg/m^2^)	6.55 ± 1.14	6.79 ± 1.03	5.54 ± 1.04	5.63 ± 1.05	< 0.001
PEF (L/min)	273.29 ± 117.03	283.27 ± 117.29	241.76 ± 106.61	203.05 ± 101.01	< 0.001
PEF%pred	79.11 ± 30.00	80.54 ± 29.66	75.60 ± 30.45	65.99 ± 30.22	< 0.001
Airflow limitation, *n* (%)	2089 (51.5)	1608 (49.3)	350 (59.1)	131 (66.2)	< 0.001

*BMI* body mass index, *HGS* hand grip strength, *5CST* five-repetition chair stand test, *SPPB* short physical performance battery, *ASM* appendicular skeletal muscle mass, *SMI* skeletal muscle mass index, *PEF* peak expiratory flow

### Cross-sectional associations of sarcopenia with PEF

Table 2 shows the associations of sarcopenia and its components with PEF and PEF%pred by linear regression analysis. After adjusting for covariates in model 3, we found that SMI, HGS, gait speed, and SPPB were positively correlated with PEF and PEF%pred, while 5CST and sarcopenia were negatively correlated with PEF and PEF%pred, respectively (all *P* < 0.01).

**Table 2:** Associations of sarcopenia and its components with PEF and PEF%pred in the cross-sectional analysis

	Model 1		Model 2		Model 3	
	β (95%CI)	*P* value	β (95%CI)	*P* value	β (95%CI)	*P* value
PEF						
HGS	5.68 (5.34, 6.02)	< 0.001	3.58 (3.15, 4.01)	< 0.001	3.13 (2.68, 3.57)	< 0.001
Gait speed	116.40 (101.10, 131.70)	< 0.001	68.51 (54.22, 82.80)	< 0.001	61.52 (47.25, 75.80)	< 0.001
5CST	− 5.85 (− 6.59, − 5.10)	< 0.001	− 3.30 (− 4.00, − 2.59)	< 0.001	− 3.07 (− 3.77, − 2.37)	< 0.001
SPPB	19.06 (17.33, 20.79)	< 0.001	11.53 (9.81, 13.25)	< 0.001	10.66 (8.91, 12.40)	< 0.001
SMI	42.27 (39.39, 45.16)	< 0.001	18.26 (13.77, 22.74)	< 0.001	69.65 (47.21, 92.08)	< 0.001
Sarcopenia	− 40.66 (− 47.33, − 33.99)	< 0.001	− 26.74 (− 33.08, − 20.40)	< 0.001	− 19.15 (− 26.69, − 11.62)	< 0.001
PEF%pred						
HGS	0.65 (0.55, 0.74)	< 0.001	0.96 (0.84, 1.09)	< 0.001	0.84 (0.71, 0.97)	< 0.001
Gait speed	18.29 (14.30, 22.28)	< 0.001	19.37 (15.27, 23.48)	< 0.001	17.51 (13.40, 21.62)	< 0.001
5CST	− 0.93 (− 1.12,− 0.73)	< 0.001	− 1.00 (− 1.21, − 0.80)	< 0.001	− 0.95 (− 1.15, − 0.75)	< 0.001
SPPB	2.98 (2.52, 3.44)	< 0.001	3.46 (2.97, 3.96)	< 0.001	3.24 (2.74, 3.74)	< 0.001
SMI	3.03 (2.22, 3.83)	< 0.001	5.24 (3.96, 6.53)	< 0.001	22.95 (16.50, 29.39)	< 0.001
Sarcopenia	− 6.36 (− 8.09, − 4.63)	< 0.001	− 7.82 (− 9.64, − 6.00)	< 0.001	− 5.82 (− 7.98, − 3.65)	< 0.001

*HGS* hand grip strength, *5CST* five-repetition chair stand test, *SPPB* short physical performance battery, *SMI* skeletal muscle mass index, *PEF* peak expiratory flow

Model 1, unadjust

Model 2, adjust for age and sex

Model 3, adjust for age, sex, education level, smoking status, drinking status, BMI, marital status, residential area, type of cooking fuels, hypertension, diabetes, heart problems, stroke, and kidney disease

We also explored the associations of sarcopenia and its components with airflow limitation by logistic regression analysis (Table S1). After adjusting for covariates in model 3, we found that compared with the non-sarcopenia group, subjects in the severe sarcopenia group were associated with a higher risk of airflow limitation (OR = 1.66, 95% CI = 1.18–2.33, *P* = 0.004). Also, higher SMI, HGS, gait speed, and SPPB were significantly associated with lower odds of airflow limitation, while the opposite pattern was found between 5CST and airflow limitation (all *P* < 0.01).

### Associations between baseline sarcopenia status and 4-year PEF decline

**Fig. 2:**
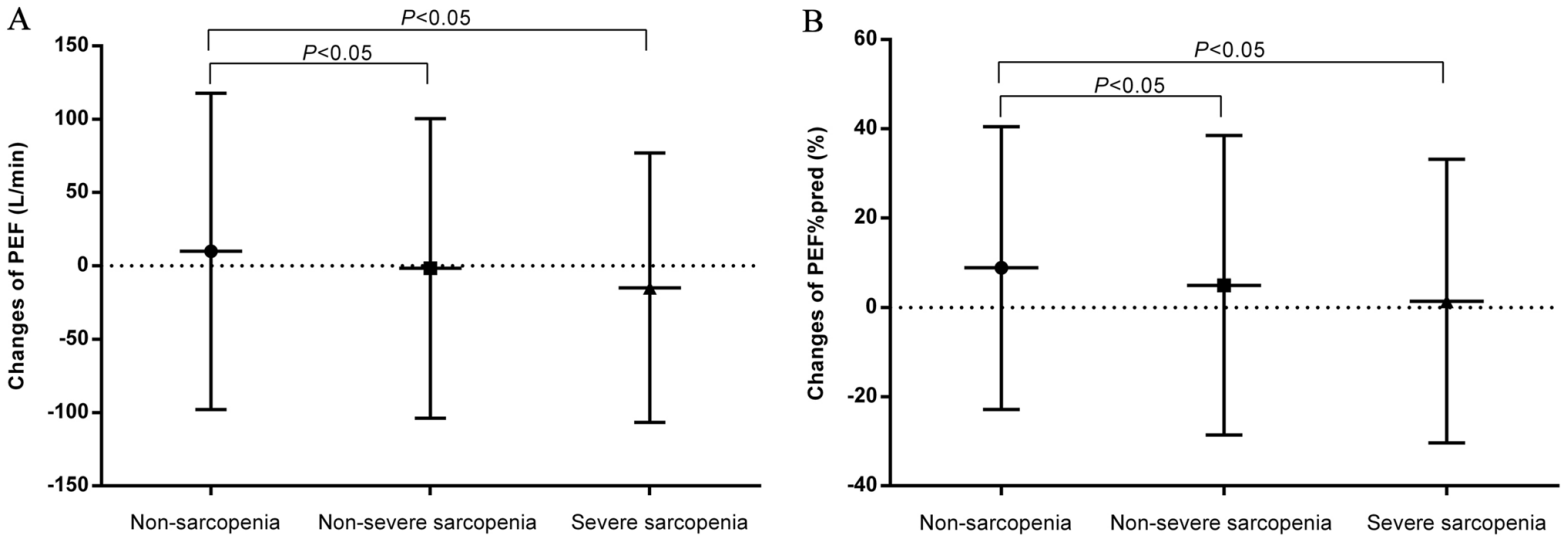
Changes in PEF (**A**) and PEF%pred (**B**) according to sarcopenia status from 2011 to 2015. Data are shown as mean ± standard deviation

Figure 2 shows the changes in PEF and PEF%pred according to baseline sarcopenia status from 2011 to 2015. We found that the changes in PEF and PEF%pred were both significantly different in the non-sarcopenia and sarcopenia groups (*P* < 0.05).

Table S2 shows the associations of the baseline sarcopenia status and its components with PEF and PEF%pred changes by linear regression analysis. After adjusting for covariates, we found that the baseline SMI, HGS, gait speed, and SPPB were positively correlated with PEF and PEF%pred changes, while sarcopenia was negatively correlated with PEF and PEF%pred changes (*P* < 0.05).

Table 3 presents the incidence of PEF or PEF%pred decline according to baseline sarcopenia status from 2011 to 2015. After adjusting for covariates in model 3, the results revealed that compared with the non-sarcopenia group, subjects in the severe sarcopenia group were both associated with a higher risk of PEF decline (OR = 2.05, 95%CI = 1.30–3.26, *P* = 0.002) and PEF%pred decline (OR = 1.83, 95%CI = 1.17–2.86, *P* = 0.008).

**Table 3:** Incidence of PEF or PEFpred% decline according to baseline sarcopenia status from 2011 to 2015

	No. of cases/total	Model 1			Model 2			Model 3	
		OR (95%CI)	*P* value		OR (95%CI)	*P* value		OR (95%CI)	*P* value
PEF decline									
No sarcopenia	1053/2299	Ref			Ref			Ref	
Non-severe sarcopenia	193/406	1.06 (0.86, 1.31)	0.568		0.98 (0.79, 1.22)	0.846		1.04 (0.78, 1.39)	0.800
Severe sarcopenia	65/115	1.53 (1.05, 2.23)	0.028		1.32 (0.89, 1.95)	0.165		2.05 (1.30, 3.26)	0.002
PEF%pred decline									
No sarcopenia	883/2289	Ref			Ref			Ref	
Non-severe sarcopenia	167/406	1.11 (0.90, 1.38)	0.330		1.03 (0.83, 1.29)	0.790		0.96 (0.72, 1.28)	0.759
Severe sarcopenia	58/115	1.62 (1.11, 2.36)	0.012		1.43 (0.97, 2.11)	0.070		1.83 (1.17, 2.86)	0.008

Model 1, unadjust

Model 2, adjust for age and sex

Model 3, adjust for age, sex, education level, smoking status, drinking status, BMI, marital status, residential area, type of cooking fuels, baseline PEF or PEF%pred, hypertension, diabetes, heart problems, stroke, and kidney disease

### Associations of the change trajectories between sarcopenic components and PEF from 2011 to 2015

Table 4 shows the associations of the change trajectories between sarcopenic components with PEF or PEF%pred from 2011 to 2015. After adjusting for covariates in model 3, we found that the change trajectories of physical performance including gait speed, 5CST, and SPPB were correlated with PEF and PEF%pred (all *P* < 0.01). The change in HGS was only significantly correlated with PEF%pred (*P* = 0.030), not with PEF (*P* = 0.056). No associations were observed between changes in SMI and PEF as well as PEF%pred.

**Table 4:** Associations of the change trajectories between sarcopenic components with PEF or PEFpred% from 2011 to 2015

	Model 1		Model 2		Model 3	
	β (95%CI)	*P* value	β (95%CI)	*P* value	β (95%CI)	*P* value
PEF change						
HGS change	0.42 (− 0.10, 0.93)	0.112	0.36 (− 0.16, 0.87)	0.176	0.41 (− 0.01, 0.82)	0.056
Gait speed change	17.80 (3.19, 32.40)	0.017	13.01 (− 1.75, 27.76)	0.084	24.96 (12.98, 36.94)	< 0.001
5CST change	− 2.32 (− 3.22, − 1.41)	< 0.001	− 2.16 (− 3.07, − 1.25)	< 0.001	− 1.92 (− 2.66, − 1.19)	< 0.001
SPPB change	4.78 (2.90, 6.65)	< 0.001	4.19 (2.30, 6.08)	< 0.001	3.66 (2.13, 5.19)	< 0.001
SMI change	− 9.12 (− 20.59, 2.35)	0.119	− 10.19 (− 21.64, 1.27)	0.081	2.22 (− 7.29, 11.74)	0.647
PEFpred% change						
HGS change	0.14 (− 0.01, 0.30)	0.070	0.13 (− 0.02, 0.29)	0.093	0.15 (0.02, 0.28)	0.030
Gait speed change	5.35 (0.96, 9.74)	0.017	4.27 (− 0.17, 8.71)	0.060	7.62 (3.85, 11.39)	< 0.001
5CST change	− 0.71 (− 0.98, − 0.44)	< 0.001	− 0.68 (− 0.95,− 0.41)	< 0.001	− 0.61 (− 0.84, − 0.38)	< 0.001
SPPB score change	1.42 (0.86, 1.98)	< 0.001	1.30 (0.74, 1.87)	< 0.001	1.16 (0.68, 1.65)	< 0.001
SMI change	− 2.35 (− 5.80, 1.10)	0.182	− 2.67 (− 6.12, 0.78)	0.130	1.07 (− 1.92, 4.07)	0.482

*HGS* hand grip strength, *5CST* five-repetition chair stand test, *SPPB* short physical performance battery, *SMI* skeletal muscle mass index, *PEF* peak expiratory flow

Model 1, unadjust

Model 2, adjust for age and sex

Model 3, adjust for age, sex, education level, smoking status, drinking status, BMI, marital status, residential area, type of cooking fuels, baseline PEF or PEF%pred, hypertension, diabetes, heart problems, stroke, and kidney disease

## Discussion

In this study, we evaluated the cross-sectional and longitudinal associations of sarcopenia and its components with pulmonary function, as assessed via PEF and PEF%pred among Chinese community-dwelling elderly people. We demonstrated that sarcopenia, skeletal muscle mass, grip strength, and physical performance were all correlated with PEF, PEF%pred, and risk of airflow limitation in the cross-sectional analysis. We also found that the individuals with severe sarcopenia were at a higher risk of incident PEF or PEF%pred decline during a 4-year follow-up. In addition, the changes in physical performance were associated with changes in PEF and PEF%pred in the longitudinal analysis. To the best of our knowledge, this is the first longitudinal study to investigate the associations of the change trajectories between sarcopenic components and PEF among Chinese community-dwelling elderly people.

Previous research on associations between sarcopenia and pulmonary function was mainly based on forced vital capacity (FVC) and FEV1 in COPD patients [8, 25, 26]. PEF is a cheap and simple screening tool for pulmonary function. According to the 2010 consensus by the European Working Group on Sarcopenia in older people, PEF is determined by the strength of respiratory muscles in people without lung diseases [1]. It can be used as an indicator of respiratory sarcopenia [15]. Recently, an increasing number of studies explored the associations between PEF and sarcopenia [27] as well as its defining components. A previous cross-sectional study including 240,562 Korean adults without clinical lung diseases demonstrated the association between PEF and skeletal muscle mass [11]. Another study focused on community-dwelling older adults reported that HGS and gait speed were more strongly associated with PEF than skeletal muscle mass [28]. In this present cross-sectional study, we added new evidence that SMI, HGS, and gait speed were associated with PEF, PEF%pred, and risk of airflow limitation.

The 5CST and SPPB are important parts of sarcopenia. It was reported that 5CTS and SPPB were responsive to pulmonary rehabilitation in patients with COPD [29]. Some previous cross-sectional studies found that abnormal SPPB was associated with abnormal pulmonary function, as assessed via FEV1 or FVC, in both COPD [30] and the aging population [31]. It is also reported by Charles et al. that compared to elderly subjects with normal PEF, subjects with reduced PEF had a lower SPPB [32]. However, the relationship between 5CST and pulmonary function remains controversial. Choi et al. found that 5CST did not exhibit a linear relationship with FEV1 and FVC [31], while Landi et al. proved that FVC, FEV1, and PEF were all linearly correlated with 5CST [33]. In our study, we provided more evidence about the relationship between SPPB and PEF, as well as 5CST and PEF.

To our knowledge, few studies have investigated the correlation of sarcopenia and its components with PEF based on longitudinal studies. Recently, a report showed that the baseline paraspinal muscle density predicted FEV1%pred decline among women with asthma [34]. In this study, we longitudinally investigate the relationship of baseline sarcopenia status with the decline of PEF during a 4-year follow-up. We found that the changes of PEF and PEF%pred were both significantly different in the non-sarcopenia and sarcopenia groups. However, just as mentioned in a previous study [35], the increase of PEF and PEF%pred during a 4-year follow-up seemed irrational in the elderly population even in the non-sarcopenia group. The reason for this increase may be associated with improved air quality and a healthy community lifestyle. The results also revealed the baseline sarcopenia status and its components including SMI, HGS, gait speed, and SPPB were all associated with PEF and PEF%pred changes, respectively. Compared with the non-sarcopenia group, subjects in the severe sarcopenia group were associated with a 2.05-times risk of PEF decline and a 1.83-times risk of PEF%pred decline.

In addition, skeletal muscle mass, grip strength, and physical performance can be changed over time. Therefore, it is necessary to examine the associations of the change trajectories between sarcopenic components with PEF. A previous longitudinal study found that an increase in skeletal muscle mass was associated with attenuated FEV1 decline in healthy men by the Pearson’s methods. Furthermore, it is proved that even prominent loss of muscle mass over time is associated with attenuated FEV1 decline in men unless it is combined with prominent gain of fat mass. The results weakened the association between muscle mass changes over time and pulmonary function change [36]. In our study, we found that the change trajectories of physical performance including gait speed, 5CST, and SPPB were associated with changes in PEF and PEF%pred over a 4-year follow-up. However, SMI did not exhibit this relationship. These differences may exist due to different populations, pulmonary function parameters, and evaluation methods for muscle mass. Our results indicated that improvement in physical performance is more important than muscle mass. As far as we know, PEF reflects respiratory muscle strength rather than respiratory muscle mass [37]. Additional studies for the relationship between pulmonary function and muscle mass changes over time are warranted.

The strengths of our study are as follows: we focused on a large elderly population from Chinese community-dwelling, and we performed both cross-sectional and longitudinal analyses to evaluate the associations of sarcopenia with PEF. However, some limitations still should be noted in our study. First, according to the 2019 consensus of AWGS, dual-energy X-ray absorptiometry (DXA) and bioelectrical impedance analysis were recommended to measure skeletal muscle mass. However, in our study, skeletal muscle mass was estimated by a previously validated anthropometric equation which may introduce some bias. According to reports in the literature, skeletal muscle mass calculated by this formula was in good agreement with DXA and widely used as a method to quantify sarcopenia [20, 38, 39]. Second, gait speed was calculated by the time to complete a 2.5-m course at a normal pace rather than a standard distance of 6-m. A systematic review including 48 studies showed that among the elderly, the walkway lengths did not produce differences in the recorded gait speed [40]. Finally, even though we have adjusted as many relevant covariates as possible in the multivariate regression analysis, there are still confounding factors that could not be completely ruled out which may contribute to a different outcome.

## Conclusions

In conclusion, we demonstrated the associations of sarcopenia with PEF as well as PEF decline in Chinese community-dwelling elderly population by cross-sectional and longitudinal analysis. We also found that changes in physical performance were associated with changes in PEF during a 4-year follow-up. Our findings suggest that improving sarcopenia, especially physical performance may contribute to the increase of PEF.

### Supplementary Information

Below is the link to the electronic supplementary material.

Supplementary file1 (DOCX 24 KB)

Supplementary file2 (DOCX 17 KB)

## Data Availability

The datasets generated and/or analyzed during the current study are available in the China Health and Retirement Longitudinal Study (CHARLS) repository, http://charls.pku.edu.cn/.
