# Supplementary material for: Associations of sarcopenia with peak expiratory flow among community-dwelling elderly population: based on the China Health and Retirement Longitudinal Study (CHARLS)
Source: Eur Geriatr Med. 2023 Jul 19;15(1):95–104. doi: 10.1007/s41999-023-00838-2 (PMC10876815; doi:10.1007/s41999-023-00838-2)
Supplement: Supplementary file 1 — Supplementary file1 (DOCX 24 KB) [file 41999_2023_838_MOESM1_ESM.docx]

**Table S1** Associations of sarcopenia and its components with airflow limitation in the cross-sectional analysis

|  | Model 1 | |  | Model 2 | |  | Model 3 | |
| --- | --- | --- | --- | --- | --- | --- | --- | --- |
|  | OR (95%CI) | *P* value |  | OR (95%CI) | *P* value |  | OR (95%CI) | *P* value |
| HGS | 0.96(0.95, 0.97) | <0.001 |  | 0.95(0.94, 0.96) | <0.001 |  | 0.95(0.95, 0.96) | <0.001 |
| Gait speed | 0.28(0.21, 0.37) | <0.001 |  | 0.29(0.21, 0.39) | <0.001 |  | 0.32(0.23, 0.43) | <0.001 |
| 5CST | 1.07(1.05, 1.09) | <0.001 |  | 1.07(1.05, 1.08) | <0.001 |  | 1.06(1.05, 1.08) | <0.001 |
| SPPB | 0.82(0.80, 0.85) | <0.001 |  | 0.82(0.79, 0.85) | <0.001 |  | 0.82(0.79, 0.85) | <0.001 |
| SMI | 0.79(0.75, 0.84) | <0.001 |  | 0.73(0.67, 0.80) | <0.001 |  | 0.29(0.18, 0.46) | <0.001 |
| Sarcopenia |  |  |  |  |  |  |  |  |
| No sarcopenia | Ref |  |  | Ref |  |  | Ref |  |
| Non-severe sarcopenia | 1.49(1.25, 1.78) | <0.001 |  | 1.51(1.26, 1.81) | <0.001 |  | 1.20(0.96, 1.50) | 0.111 |
| Severe sarcopenia | 2.01(1.49, 2.72) | <0.001 |  | 2.14(1.56, 2.92) | <0.001 |  | 1.66(1.18, 2.33) | 0.004 |

HGS, hand grip strength; 5CST, five-repetition chair stand test; SPPB, short physical performance battery; SMI, skeletal muscle mass index; PEF, peak expiratory flow.

Model 1, unadjust.

Model 2, adjust for age and sex.

Model 3, adjust for age, sex, education level, smoking status, drinking status, BMI, marital status, residential area, type of cooking fuels, hypertension, diabetes, heart problems, stroke, and kidney disease.
