# Supplementary material for: Associations of sarcopenia with peak expiratory flow among community-dwelling elderly population: based on the China Health and Retirement Longitudinal Study (CHARLS)
Source: Eur Geriatr Med. 2023 Jul 19;15(1):95–104. doi: 10.1007/s41999-023-00838-2 (PMC10876815; doi:10.1007/s41999-023-00838-2)
Supplement: Supplementary file 2 — Supplementary file2 (DOCX 17 KB) [file 41999_2023_838_MOESM2_ESM.docx]

**Table 2S** Associations of the baseline sarcopenia status and its components with PEF and PEF%pred changes from 2011 to 2015

|  | PEF change | |  | PEF%pred change | |
| --- | --- | --- | --- | --- | --- |
|  | β (95%CI) | *P* value |  | β (95%CI) | *P* value |
| HGS | 1.53(1.06, 2.00) | <0.001 |  | 0.45(0.30, 0.59) | <0.001 |
| Gait speed | 18.57(4.14, 33.00) | 0.012 |  | 5.84(1.36, 10.31) | 0.011 |
| 5CST | -0.47(-1.27, 0.33) | 0.249 |  | -0.21(-0.46, 0.04) | 0.098 |
| SPPB | 1.94(0.04, 3.85) | 0.046 |  | 0.79(0.20, 1.38) | 0.009 |
| SMI | 35.97(12.71, 59.22) | 0.002 |  | 12.46 (5.25, 19.67) | 0.001 |
| Sarcopenia | -13.29(-21.15, -5.42) | 0.001 |  | -4.29 (-6.73, -1.86) | 0.001 |

HGS, hand grip strength; 5CST, five-repetition chair stand test; SPPB, short physical performance battery; SMI, skeletal muscle mass index; PEF, peak expiratory flow.

Adjust for age, sex, education level, smoking status, drinking status, BMI, marital status, residential area, type of cooking fuels, baseline PEF or PEF%pred, hypertension, diabetes, heart problems, stroke, and kidney disease.
